# The Effect of Atorvastatin on Oncogenic miRNAs in Hematological Malignancies: A Central Study

**DOI:** 10.3390/biom14121559

**Published:** 2024-12-07

**Authors:** Jood Hashem, Farah Alsukhni, Hassan Abushukair, Mahmoud Ayesh

**Affiliations:** 1 Department of Medical Laboratory Sciences, Jordan University of Science and Technology, Irbid 22110, Jordan; 2 Faculty of Medicine, Jordan University of Science and Technology, Irbid 22110, Jordan

**Keywords:** statin, hematological malignancies, microRNA, lymphoma, leukemia

## Abstract

The efficacy of statins as anti-cancer drugs has been demonstrated in several malignancies but has been poorly investigated in hematological malignancies (HM). By studying its effect on oncogenic miRNAs, we investigated the effect of statin therapy on HM patients. The data were used to identify enriched pathways that were altered due to statin treatment. The main aim of this study was to identify significantly differentially expressed miRNAs and involved regulatory pathways post-atorvastatin treatment in HM patients. A panel of 95 plasma circulating miRNAs involved in tumorigenesis, apoptosis, and differentiation were relatively quantified using qPCR for blood samples obtained from 12 HM patients, 4 with Chronic Myeloid Leukemia (CML), 4 with Non-Hodgkin Lymphoma (NHL), and 4 with Essential Thrombocythemia. Pre- and post-administration of a 6-week atorvastatin course miRNA expression levels were measured. Significantly differentially expressed miRNAs were those with a fold change >2 or <0.5 using the Livak method with a two-sided *p*-value < 0.05. To further understand the underlying mechanism of statin regulated miRNA, GO and KEGG pathway enrichment analyses were conducted for identified target genes using the DAVID 6.8 bioinformatics tool. Out of 95 miRNAs, 14 exhibited significant fold changes post-treatment with statins including miR-198, miR-29a+b+c, miR-204, miR-222, miR-224, miR-155, miR-128b, miR-296, miR-199a+b, miR-194, miR-125a, miR-200a, and the let-7-family that were upregulated and miR-150 that was downregulated post-statin treatment. Higher mir-222, mir-194, mir-128b, and mir-199b expressions were significantly associated with better overall survival using the Cancer Genomic Atlas leukemia and lymphoma patient cohorts. Enrichment analysis identified the PI3k-Akt pathway as well as other pathways involved in the epithelial–mesenchymal transition. Atorvastatin upregulated several tumor suppressor genes involved in mediating better prognosis. The data can be used to enhance personalized treatments for patients with hematological malignancies by helping to predict the different pathways that may be targeted and, therefore, result in better survival outcomes in these patients.

## 1. Introduction

Hematological malignancies (HMs) are tumors that target blood-forming tissues or cells in the immune system [1]. It has become clearer that these malignancies include a very large number of genetically diverse diseases [2]. HMs account for 10% of all cancers worldwide with a 60% five-year survival rate [3]. Survival rates have been increasing with emerging new therapies that use combined mechanisms to target different HM. Immunotherapy [4,5], personalized target therapy [6,7], chemotherapy [8], and bone marrow [9] transplantation are all among the common therapies used to treat HM to date. Despite the evident progress, HM patients still face major challenges that impede the success rates of these treatment regimens, including the relapse of a more aggressive form of the disease [10], metastasis, and drug resistance [11].

Recently, several studies have explored the use of statins as adjuvant therapy, to enhance the effectiveness of chemotherapy and improve the status of the immune system [12,13,14]. Statins, the 3-hydroxy-methylglutaryl coenzyme A (HMG-CoA) reductase inhibitors, are commonly used as potent inhibitors for cholesterol synthesis. Particularly, statins are essential for the treatment of lipid disorders and for cardiovascular disease prevention [15]. Convincing evidence has demonstrated the pleiotropic effects of reducing the cellular cholesterol precursor, mevalonic acid, on improving the immune system and cancer prevention in prostate, lung, colon, and breast cancer [16,17]. In HM specifically, studies have reported that statin users have a 24% decreased risk factor, especially for long-term users [18,19]. However, contrary to the original understanding of the biological effect of statins, recent studies have shown that the benefits of statins are not solely mediated by their lipid-lowering properties [20]. It has become evident that statin treatment could also influence cellular and molecular pathways that mediate tumor characteristics. Nevertheless, predicting the effect of statins on these pathways remains a challenge in several tumors.

Current methods that are mainly used to diagnose, guide the treatment process, and predict the survival rates among HM patients include bone marrow and/or tissue aspiration and biopsy. These methods are considered painful and invasive procedures. Yet, they are considered the gold standard for diagnosing HM. However, several studies have recently begun to consider microRNAs (miRNAs) as new potential noninvasive biomarkers for diagnosing cancer and other diseases [14]. miRNAs are small (less than 22 nucleotides) non-coding RNAs that play an important role in regulating gene expression by interacting with the 3′ untranslated regions (UTRs) of target mRNAs, leading to their premature degradation and translational suppression [21]. miRNA’s shuttling mechanism is mediated by extracellular vesicles known as exosomes. Exosomes travel through the bloodstream, transporting miRNAs that repress the mRNA when they reach their target cells.

Changes in the expression of miRNAs have been found to influence the regulation of significant pathological processes in tumor formation as well as their progression and metastasis [22,23,24,25,26]. Differential miRNA expression has been depicted in various types of cancers such as breast, pancreatic, lung, and colorectal cancer [27,28,29,30]. The dysregulation of miRNA biogenesis pathways has also been linked to HM. Just like in several other tumors, miRNAs in HM can act as oncogenes such as miR-150, miR-130, miR-331, miR-221, and miR-96 or tumor suppressors such as miR-198, miR-29, let-7 family, and miR-151 and their aberrant expression can mediate drug resistance (miR-221, miR-222, miR-155, miR-128a, and several others) [31,32,33,34]. As a result, several studies have suggested the use of miRNAs as biomarkers for drug response and clinical outcome prediction, as well as promising therapeutic tools in hematological diseases [34,35,36].

Due to the pleiotropic effects of statins in tumors and their ability to modulate molecular pathways, in this study, we chose to examine the effect of statins on miRNA modulation in HMs. Although several studies have shown the effect of statins on several tumors, the amount of data on their influence on different HMs is small. To our knowledge, this is the first study that shows the effect of statins on different miRNAs in hematological malignancies. Using a panel of miRNAs, in this study, we examined the effect of statins on these miRNAs in HM patients and used the data to predict the different pathways that are influenced by this treatment.

## 2. Materials and Methods

This manuscript was written in accordance with the Strengthening the Reporting Of Pharmacogenetic Studies (STROPS) guidelines [37].

### 2.1. Participants

In this single-institute interventional study, we included patients attending hematology clinics at the King Abdullah University Hospital with hematological malignancy. HM represented in this study included Essential Thrombocythemia (ET), Chronic Myeloid Leukemia (CML), and Hodgkin Lymphoma (HL). Patients had to be compliant with their cancer treatments and were not on any statin therapies, specifically atorvastatin, in order to be eligible for inclusion. Patients were excluded if they had one of the following: (1) end-stage malignancies, (2) on statin prior to the start of the study, (3) a history of recent acute illness or clinical evidence indicative of myopathies and gastropathies, and (4) statin contraindications. The recruitment period lasted 12 months (December 2017 to December 2018) and clinical follow-up of included patients was achieved through the patient’s medical record. All patients were on their regular treatment regimen throughout the study. ET patients took hydroxyurea and CML patients were on imatinib. Hodgkin Lymphoma patients were on doxorubicin, bleomycin sulfate, vinblastine sulfate, and dacarbazine. Controls on a similar atorvastatin course (40 mg/day for 6 weeks) without any history of malignancies were included in this study.

This study was approved by the Institutional Review Board (IRB) committee (290/2016) at the Jordan University of Science and Technology (JUST). Written informed consent was obtained from each participant before enrollment in the study.

### 2.2. Intervention and Sample Collection

For all enrolled patients, two blood samples were taken during the study: the first at the baseline upon recruitment and the second after one month of the atorvastatin course (40 mg/day for 6 weeks). Peripheral venous blood was collected after an overnight fast into two tubes, an evacuated Ethylenediaminetetraacetic acid (EDTA) tube (AFCO, Jordan, 5 mL of blood), and a plain tube (AFCO, Jordan, 10 mL blood) to measure onco-miRNAs expression and exosomes abundance, respectively.

The serum was separated after centrifuging blood samples in plain tubes at 4000× *g* for 5 min at 4 °C within 30 min of collection. Then, serum samples were aliquoted in two Eppendorf tubes and stored at −80 °C until exosome quantification. Blood in EDTA tubes was immediately centrifuged at 3500× *g* for 10 min at 4 °C, then plasma samples were aliquoted in two Eppendorf tubes and re-centrifuged at 5000× *g* for 15 min at 4 °C to 21 remove any remaining cell debris. Trizol was then added to the supernatant and stored at −80 °C for further analysis.

### 2.3. Molecular Study

#### 2.3.1. miRNA Extraction and cDNA Synthesis

The Qiagen miRNeasy Serum/Plasma kit (Qiagen; Hilden, Germany cat# 217184) was used to extract miRNAs from plasma. The procedure was performed using standard phenol/guanidine lysis of the samples and silica-membrane-based purification using RNeasy MinElute spin columns. Complementary DNA (cDNA) was synthesized using QuantiMir Kit (System Biosciences; Palo Alto, CA, USA cat# RA420A-1).

#### 2.3.2. miRNA Quantification by Real-Time qPCR

Circulating miRNAs were quantified after cDNA synthesis by real-time quantitative PCR (rt-qPCR) using an Applied Biosystems 7500 Fast-well system (Applied Biosystems, Warrington, UK). The quantitative assay was performed by using the OncoMir qPCR Array (System Biosciences; CA, USA) based on the amplification of a panel of 95 miRNAs involved in tumorigenesis, apoptosis, and differentiation (Table S1). Specific miRNA primer sequences and the universal miRNA sequences were previously designed by the manufacturer. The relative expression level difference between the four groups in our study (pre- and post-atorvastatin administration for both HM patients and control subjects was conducted by comparing cycle threshold CT values using the Livak method (2^−∆∆CT^) for relative quantification [38].

#### 2.3.3. Exosome Precipitation and Quantification

Circulating plasma exosome isolation was performed using a solution containing a CD63 binding polymer, which is an exosome-specific marker. This resulted in the precipitation of exosomes between 30 and 200 nm in size from serum samples. Exosomes were quantified by ELISA using the ExoELISA-ULTRA Complete Kit (CD63 detection, System Biosciences). Details on cDNA synthesis, rt-qPCR reaction setup, and exosome precipitation and concentration are provided in the Supplementary Material File.

#### 2.3.4. Differentially Expressed miRNA Gene Target Prediction

Differentially expressed miRNAs (DEMI) were defined as those with a fold change greater than 2 with a statistically significant *p*-value (<0.05) for pre- and post-miRNA expression. In order to further understand DEMIs’ effect on the molecular level, gene target prediction analysis was conducted. DEMI targets were predicted using three online analysis tools, including (1) miRDB (http://www.mirdb.org/ accessed on 1 June 2022) from which we only included genes with a target score ≥ 60, (2) TargetScanHuman from which we only included genes with a total context score ≤ −0.2 (http://www.targetscan.org accessed on 1 June 2022), and (3) miRTarBase (https://mirtarbase.cuhk.edu.cn/~miRTarBase/miRTarBase_2022/php/index.php accessed on 1 June 2022).

#### 2.3.5. Functional Enrichment Analyses

To conduct the enrichment analysis, functional annotation terms for Gene Ontology and Kyoto Encyclopedia of Genes and Genomes were extracted using the DAVID 6.8 bioinformatics database [39].

#### 2.3.6. Survival Analyses

Survival analysis was conducted using the Acute Myeloid Leukemia (AML) and Diffuse Large B-Cell Lymphoma (DLBCL) cancer genomic atlas (TCGA) cohorts [40].

#### 2.3.7. Statistical Analysis

Quantitative data are presented with a mean ± standard deviation. A paired sample *t*-test was used to assess fold change significance. A One-way Analysis of Variance (ANOVA) test was used to compare pre- and post-treatment miRNA expression across different disease subgroups, and if this resulted in a significant difference, post-hoc analysis would include Tukey’s multiple comparison tests to compare disease pairs. On the patient level, the Wilcoxon Signed-Rank test was used to assess the miRNA mean difference statistical significance. For functional enrichment analysis, Fisher’s exact test was used to assess the overrepresentation of a certain category; the resulting *p*-value was corrected for multiple testing using the Benjamini–Hochberg false discovery rate (FDR) method. A two-sided *p*-value of <0.05 was considered statistically significant. Statistical Package for Social Sciences (SPSS) version 22.0 (SPSS, Inc., Chicago, IL, USA) was used for statistical analyses [41], and GraphPad Prism version 9.2.0 for Windows was used to design figures [42].

## 3. Results

### 3.1. Patients’ Characteristics

**A total of 12 patients with hematological malignancies were enrolled in this study.** Those included four patients with CML, four with ET, and four with HL. The mean age of the participants was 56.3 (±5.6) with a 50:50 gender representation. Complete blood count measurements for patients pre- and post-treatment with atorvastatin (Table 1) show that there were no significant differences due to treatment.

### 3.2. Atorvastatin Effect on Oncogenic miRNA Expression

**Figure 1:**
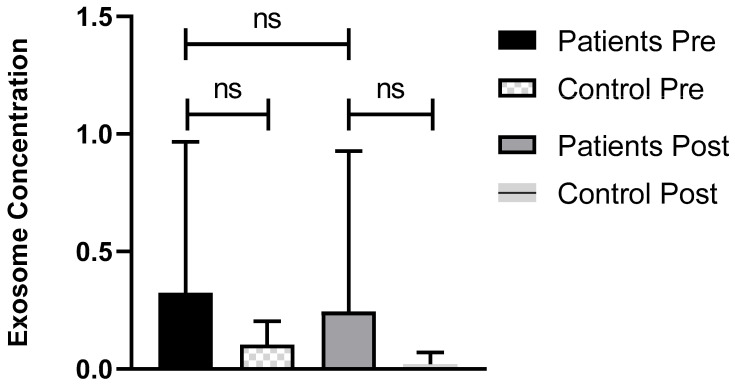
The effect of atorvastatin on serum exosome concentration in hematological malignancies patients (n = 12) and controls (n = 4). Mean exosome concentration in included patients was 0.32 (± 0.64) pre-treatment and 0.24 (± 0.68) post-treatment; there was no statistically significant difference between both settings (*p* = 0.73). While in controls, the pre-treatment concentration was 0.10 (± 0.09) and 0.02 (± 0.05), in post-treatment there was no significant difference (*p* = 0.37). No significant difference was found between controls and patients in exosome concentration (*p* = 0.51). Abbreviations—ns: not significant.

Figure 1 shows that there was no statistically significant difference in the exosome concentration in HM patients pre- and post-atorvastatin treatment (0.32 vs. 0.24, *p* = 0.73). Also, there was no significant difference between patients and controls pre-treatment (0.32 vs. 0.10, *p* = 0.37).

**Figure 2:**
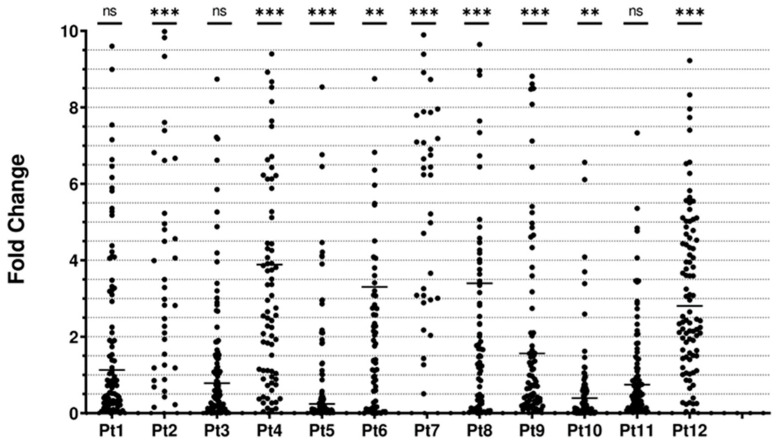
miRNAs fold changes in hematological malignancies patients. Each dot demonstrates one of the investigated 95 miRNAs involved in tumorigenesis, apoptosis, and differentiation. Horizontal lines for each patient represent the median fold change. *p*-values were calculated using the Wilcoxon Signed-Rank test for mean difference (ns: not significant, **: *p* < 0.005, and ***: *p* < 0.0005).

Subsequently, the differential expression of oncogenic miRNAs in each patient was assessed pre- and post-treatment with atorvastatin. A panel of 95 oncogenic miRNAs was tested and all patients but three (two with ET and one with CML) exhibited a significant change in the total 95 miRNA expression levels post-treatment with atorvastatin (Figure 2 and Table 2). There was a general increase in the miRNA expression after treatment with atorvastatin in all patients except for patients (Pt3, Pt5, Pt10) who showed a slight decrease in the miRNA expression instead (Figure 2 and Table 2).

**Figure 3:**
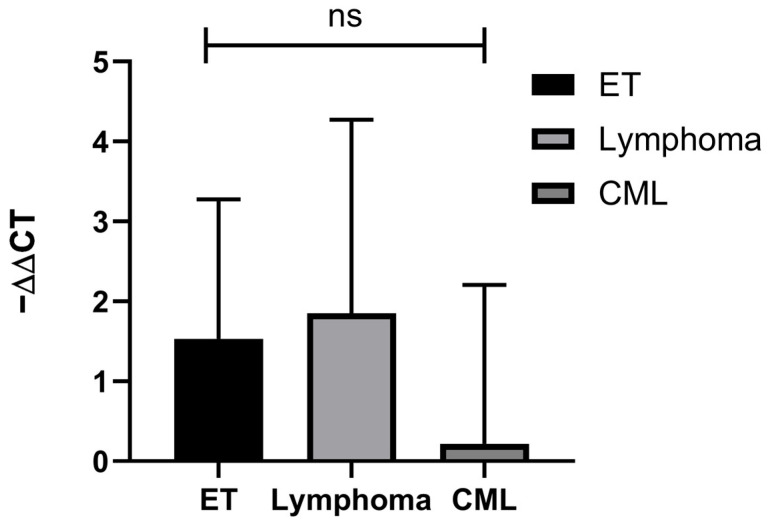
Comparison of mean −ΔΔCT (ΔCT_Post_—ΔCT_Pre_) across disease subgroups: ET = −1.53 (SD = 1.75, n = 4), Lymphoma: −1.85 (SD = 2.42, n = 4), CML: −0.22 (SD = 1.99, n = 4). One-way ANOVA for mean ΔΔCT was statistically insignificant across all groups (*p*-value = 0.522). Abbreviations—ET: Essential Thrombocythemia, CML: Chronic Myeloid Leukemia, ns: not significant.

The differential expression of miRNA was also assessed and compared in different disease subgroups to understand if the prior differences observed were relevant to a certain disease subgroup. However, no statistically significant difference was found between the different HM subgroups post-treatment with atorvastatin (*p* = 0.522, Figure 3).

### 3.3. Differentially Expressed miRNAs (DEMIs)

**Figure 4:**
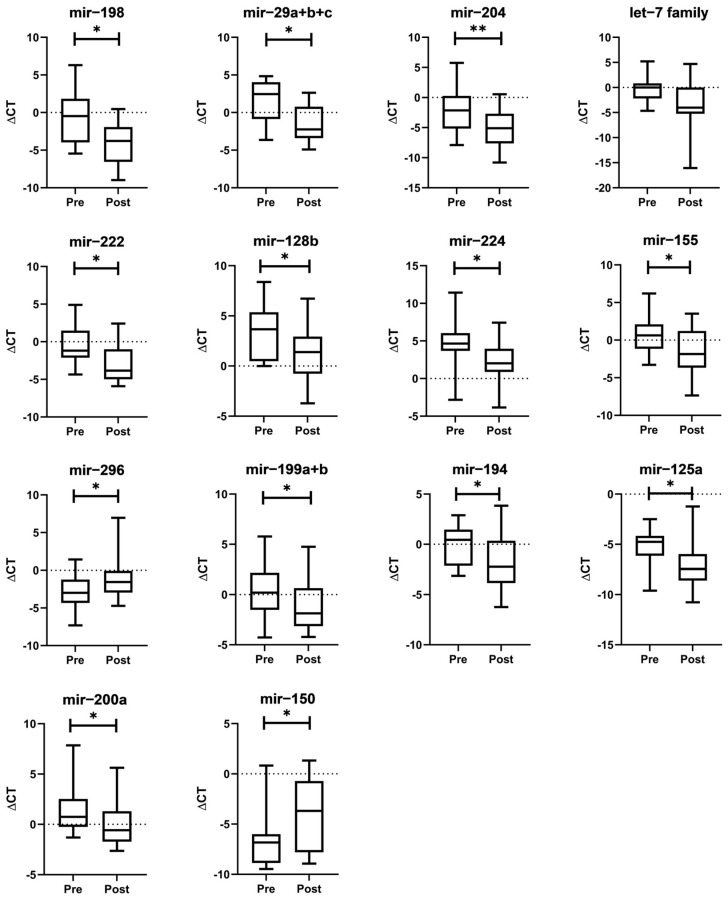
Box plots comparing pre- and post-treatment ΔCT (CT_target miRNA_—CT_control miRNA_) in significant differentially expressed miRNAs in hematological malignancy patients (n = 12). This was defined as a miRNA with a fold change ≥2 or ≤0.5 with a two-sided *p*-value ≤ 0.05. Thirteen miRNAs met this cut-off point, with all being upregulated post-treatment except for one (mir-150), which was downregulated post-treatment. (*: *p* ≤ 0.05 and **: *p* < 0.005).

Out of 95 oncogenic miRNAs tested in this study, 13 miRNAs demonstrated significant expression alterations (fold change > 2, *p* < 0.05) post-treatment with atorvastatin across all included patients, as shown in Table 3 and Figure 4. Those were miR-198, miR-29a+b+c miR-204, miR-222, miR-224, miR-155, miR-128b, miR-296, miR-199a+b, miR-194, miR-125a, miR-200a, and miR-150. All these miRNAs were significantly up-regulated post-treatment with atorvastatin except for miR-150, which was significantly down-regulated instead. The let-7 family miRNA, although very close, did not make the cut-off needed for significance (*p* = 0.0557); it was included in the table due to the major 9.6-fold change observed. Contrary to HM patients, control patients showed no significant fold change on the levels of miRNA post-treatment with atorvastatin, especially those that were found altered in HM patients (Table S2), indicating that the statin effect was specifically pertinent to miRNAs in patients with HM. The remaining 81 miRNAs with non-significant fold changes are included in Table S3.

**Figure 5:**
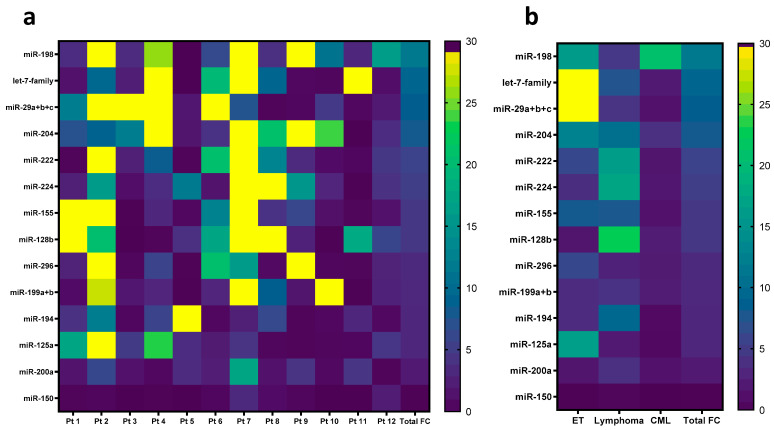
Heatmap illustrating fold changes in significant differentially expressed miRNAs (**a**) across hematological malignancy patients (n = 12) and (**b**) across disease subgroups: ET (n = 4), Lymphoma (n = 4), CML (n = 4). Bright yellow cells indicate the highest fold change whereas dark purple cells indicate the lowest fold changes. Abbreviation—ET: Essential Thrombocythemia, CML: Chronic Myeloid Leukemia, FC: fold change.

Subsequently, the differential expression of 14 miRNAs was illustrated in a heatmap depicting the fold change in each miRNA in every patient (Figure 5a) and in every represented HM category (Figure 5b). The data show that the highest miRNA fold changes were observed in patient 2 (ET) and patient 7 (HL). In addition, the most substantial miRNA fold change was observed in ET when compared to CML and Lymphoma. Intriguingly, miR204, miR29a+b+c, let-7 family, and miR-198 commonly exhibited the highest fold changes among patients 2 and 7 and in ET.

### 3.4. Functional Enrichment Analysis for Target Genes

**Figure 6:**
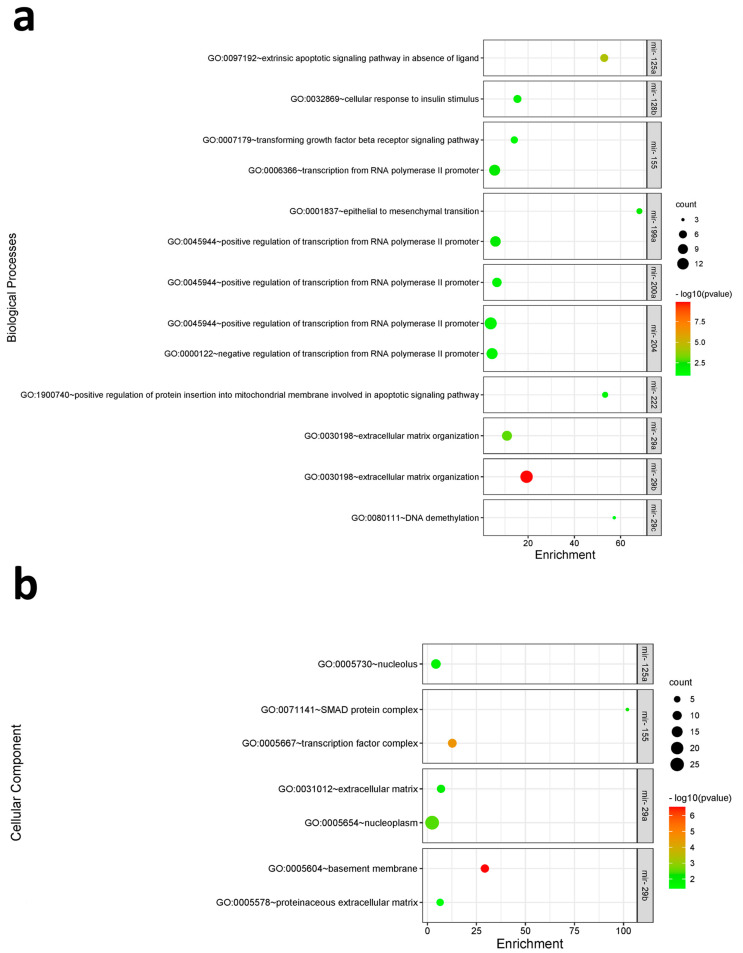

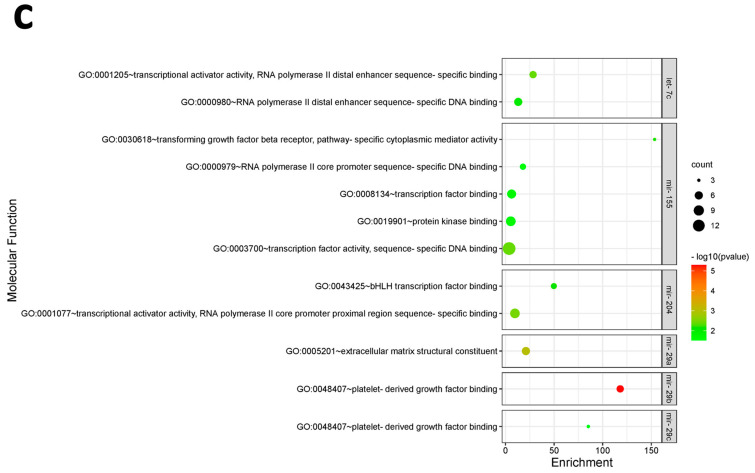
Bubble charts demonstrating enriched Gene Ontology terms for genes targeted by differentially expressed miRNAs. Enriched (**a**) biological processes, (**b**) cellular components, and (**c**) molecular functions terms are shown on the Y-axis, whereas the X-axis represents the fold enrichment level for each term. The color and size of the bubble represent the number of genes involved in each GO term and significance, respectively. An adjusted Benjamini *p*-value < 0.05 was considered significant.

**Figure 7:**
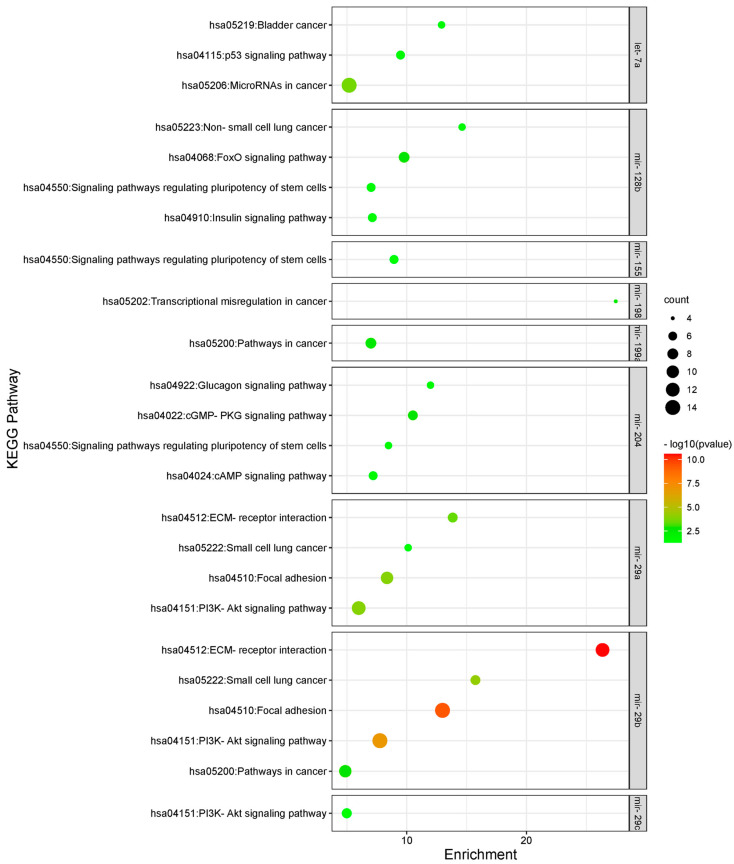
Bubble charts demonstrating enriched KEGG Pathways (on the Y-axis) in predicted target genes for differentially expressed miRNAs. The X-axis represents the fold enrichment level for each term. The color and size of the bubble represent the number of genes involved in each GO term and significance, respectively. An adjusted Benjamini *p*-value < 0.05 was considered significant.

To understand the cellular and molecular functions affected by the differential expression of the different miRNAs in our study further, a functional enrichment analysis for their target genes was conducted using the DAVID 6.8 bioinformatics functional annotation database [40]. In this study, the Gene Ontology analyses were included to show the three classes explaining the biological function of gene sets at different levels, i.e., the biological process (BP), cellular component (CC), and molecular function (MF). The biological processes enriched in target genes included the “extrinsic apoptotic signaling pathway in absence of a ligand”, “transforming growth factor beta receptor signaling pathway”, and “epithelial to mesenchymal transition” (Table S4 and Figure 6a). For cellular components, there was enrichment for “extracellular matrix”, “SMAD protein complex”, and “nucleoplasm” (Table S5 and Figure 6b). As for molecular functions, the enriched terms included “platelet-derived growth factor binding”, “bHLH transcription factor binding”, and “transforming growth factor beta receptor, pathway-specific cytoplasmic mediator activity” (Table S6 and Figure 6c). Lastly, the Kyoto Encyclopedia of Genes and Genomes (KEGG) signaling pathway enrichment analysis was conducted to illustrate the underlying oncogenic pathways affected by the target genes, which included “p53 signaling pathway”, “PI3K-Akt signaling pathway”, “cGMP-PKG signaling pathway”, “Signaling pathways regulating pluripotency of stem cells”, and “Focal adhesion” (Table 4 and Figure 7).

### 3.5. Survival Data

**Figure 8:**
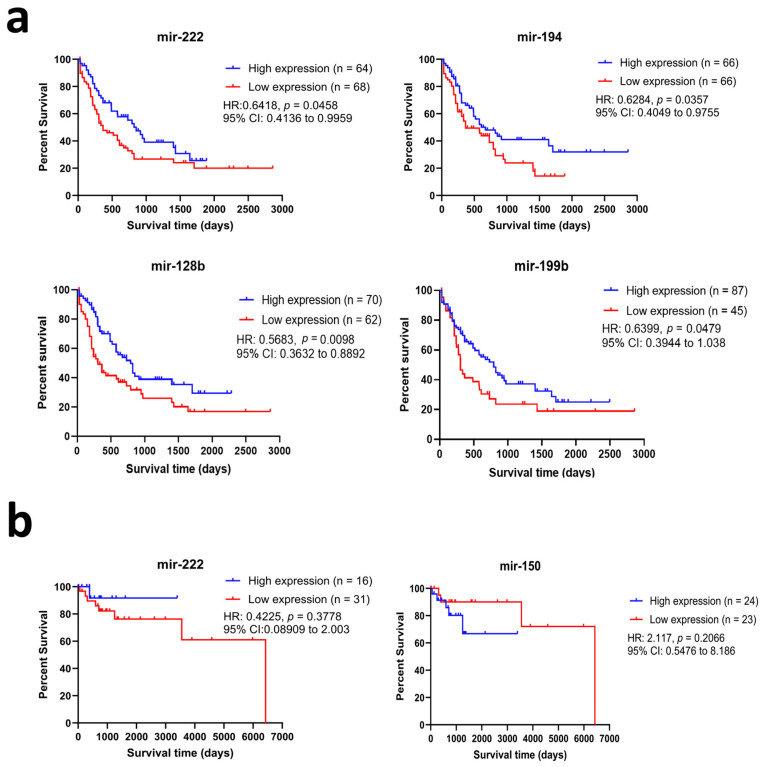
Overall survival curves demonstrating the prognostic value of significant differentially expressed miRNAs in (**a**) Acute Myeloid Leukemia (AML) patients and (**b**) Diffuse Large B-Cell Lymphoma (DLBCL) patients from The Cancer Genomic Atlas. RNA-seq data for available miRNAs, overall survival status, and period for AML and DLBCL patients were retrieved from the TCGA-AML and TCGA-DLBCL projects using the web-based tool UCSC Xena (https://xena.ucsc.edu/ accessed on 1 June 2022). Patients were stratified into high- and low-expression groups using the median expression as a cutoff point. Overall survival curves were plotted using the Kaplan–Meier method and a comparison between high- and low-expression cohorts was conducted using the log-rank test. Abbreviations—HR: Hazard Ration, CI: Confidence Interval.

For prognostic analysis purposes, overall survival data for HM patients were retrieved from the TCGA-AML and TCGA-DLBCL projects. Patients were stratified into high- and low-expression groups using the median expression as a cut-off point. From DEMI retrieved through the TCGA database, higher mir-222, mir-194, mir-128b, and mir-199b expressions were significantly associated with better chances of survival in the AML cohort (Figure 8a). As for DLBCL, there were no significant associations, yet it is worth noting that mir-150 demonstrated a trend toward better survival in patients with low expression (Figure 8b).

## 4. Discussion

To our knowledge, this is the first study to systematically assess oncomirs’ levels pre- and post-statin therapy in hematological malignancies. We report a significant upregulation in 12 miRNAs (miR-198, miR-29a+b+c miR-204, miR-222, miR-224, miR-155, miR-128b, miR-296, miR-199a+b, miR-194, miR-125a, miR-200a) and downregulation in one miRNA (mir-150).

Statin’s role in cancer has been the topic of many cancers interventional and epidemiological studies. The exact mechanism behind the effect of statins on cancer remains unclear, yet there is evidence that suggests the role of statins in inhibiting cell growth, promoting apoptotic cell death, and inhibiting matrix metalloproteinases, which are involved in invasion and metastasis [43,44,45]. While previous studies have mainly focused on the clinical benefit of statins in reducing recurrence and long-term mortality, no studies have addressed the epigenetic modulatory role of statins. This is a relevant perspective since prior studies have elucidated the role of statin in miRNA levels in controlling cholesterol levels and preventing cardiovascular disease [16,46].

With the growing shift in oncology toward a precision perspective on cancer management, miRNAs are vital in understanding the biology of cancers and how they respond to treatment. miRNA’s potential role in cancer is owed to their involvement in cell survival and apoptosis via regulating the expression of tumor suppressor genes and oncogenes [47]. Beyond involvement in cancer development, miRNAs are thought to provide substantial diagnostic value as in colorectal cancer [48], as well as a promising role in treatment delivery [49,50]. Furthermore, miRNA targeting therapies are emerging as a promising option in certain cancers [51]. Consequently, an understanding of miRNA dynamics in the context of cancer therapy will further catalyze the quest for optimal therapeutics.

In our study, mir-29a+b+c were among the significantly highly upregulated miRNAs as their targeted genes were found to be involved in extracellular matrix organization and had significant enrichment of crucial cancer-related pathways such as the PI3K-AKT signaling pathway (Figure 6 and Figure 7). Previous studies have also pointed out a tumor suppressor role for mir-29b in HM specifically DLBCL, mantle cell lymphoma, and B-cell lymphomas in general by targeting the oncogenic transcription factor E2F1 [36]. Mir-29a was also found to increase sensitivity to antineoplastic agents in leukemic cells [52]. Another important up-regulated miRNA in our cohort was mir-204, which was found to have a role in basic Helix-Loop-Helix (bHLH) transcription factor binding (Figure 6). This superfamily is involved in the regulation of the cell cycle as well as many developmental processes including cardiovascular development, hematopoiesis, and stem cell maintenance [53]. Lower expression of miR-204 was correlated with shorter survival in patients with AML [34,36]. On the contrary, its higher expression was found to be linked to pro-apoptotic mechanisms and the suppression of self-renewal of cancer stem cells [54].

Mir-155, being one of the most investigated miRNAs in HM, was found to be up-regulated in our sample and through functional analysis was found to have a role in the transforming growth factor beta receptor signaling pathway (Figure 6). In this regard, TGF-β has a dual role in cancer according to the stage of tumor formation, as in the earlier pre-malignant stages it exhibits a suppressive role by including cell cycle arrest and apoptosis while at later stages of progression, tumor cells become resistant to it and paradoxically secreted TGF-β enhances immunosuppression and enhances tumor angiogenesis, invasion, and metastasis [55,56,57]. High miR-155 expression levels down-regulated genes such as Myb and Kit, which were found to be essential in the proliferation and tumor development in AML [58]. In adult T-cell leukemia/lymphoma, high mir-155 expression was also found to correlate with longer overall survival [59]. On the contrary, its downregulation has also been shown to contribute to Vincristine resistance in DLBCL [60].

On the contrary, miR-150, a very well-studied oncogene, has been shown to play a significant role in mediating tumorigenesis in CLL, AML, MDS, and Burkitt’s lymphoma [36]. miR-150 has been shown to be down-regulated upon treatment with statin in HM patients. In CLL, mir-150 was found to be highly expressed in patients’ serum and was associated with poor prognosis [61]. The data indicate that its down regulation would lead to better survival in HM, as shown in Figure 8. Also, in our TCGA exploratory analysis, despite being statistically insignificant, our survival analysis showed a trend toward better survival in patients with lower mir-150 expression. The identified important targets of mir-150 in our in silico analysis and previous studies include MYB, FLT3, CBL, EGR2, and AKT2. The growing evidence on the oncogenic role of mir-150 opens the door for potential antagonistic therapeutic approaches in the future.

Another upregulated miRNA in our cohort, mir-224, is known to reduce resistance to imatinib in CML [62]. The Let-7 family major tumor suppressor, post-statin treatment, has been up-regulated around 10-fold. In this study, we show that statins induced the upregulation of these genes, which indicates less chemo-resistance and better prognostic and survival inclinations given the mechanistic and survival data available in different databases.

For the survival impact of miRNA levels in HM patients, mir-222, mir-194, mir-128b, and mir-199b were all significantly associated with better survival probability in AML patients. Interestingly, all these miRNAs were significantly upregulated in our sample post-statin treatment. Yet, it is important to consider the clinical and molecular differences between AML and HM included in our sample when interpreting these results. In AML for instance, mir-128 overexpression was found to decrease the viable cell number and increase doxorubicin and etoposide sensitivity in cell lines [63].

Despite the promising findings of this study, the limited sample size and restricted clinical data prevent comprehensive statistical analyses such as multivariate regression or repeated measures ANOVA. These methods, while valuable for exploring relationships between miRNA expression and clinical characteristics or temporal changes, require larger cohorts and more detailed clinical information. Additionally, while cluster analysis could have identified expression patterns among patients, our focus was on individual miRNAs to provide a clearer understanding of their functional implications. Also, the underlying molecular and clinical heterogeneity between the included three HMs (CML, ET, and HL) are well described and could impact our findings. Lastly, target genes were not measured in our experiment; hence, mechanistic insight was limited to in silico analysis and was not validated in our patients. Therefore, future controlled trials on large samples are needed to validate our results and correlate them with relevant clinical outcomes.

## 5. Conclusions

We were able to identify significant differential expression in 13 oncomirs, of which 12 miRNAs had highly differential upregulation (miR-198, miR-29a+b+c miR-204, miR-222, miR-224, miR-155, miR-128b, miR-296, miR-199a+b, miR-194, miR-125a, miR-200a) and downregulation in one miRNA (mir-150). Further investigations of the exact mechanism of how statins can affect those miRNA levels in HMs as well as mechanistic insight into the function of each miRNA in such settings are needed.

## Figures and Tables

**Table 1:** Patients’ characteristics and complete blood count measurements pre- and post-atorvastatin treatment. No significant difference was found in patients pre- and post-treatment. Abbreviations—SD: Standard Deviation, Hb: Hemoglobin, Ht: Hematocrit, WBCs: White Blood Cells, RBCs: Red Blood Cells.

	Pre-Treatment—Mean (±SD) n = 12	Post-Treatment—Mean (±SD) n = 12	*p*-Value
**Age (years)**	56.3 (5.6)		
**Sex**	M: 6, F: 6		
**Hb (g/dL)**	13.1 (1.8)	12.8 (1.8)	0.6870
**Ht (%)**	40.3 (5.9)	38.8 (5.6)	0.5296
**Cell Count (10^9^/L)**			
**WBCs**	8.5 (5.3)	8.1 (4.4)	0.8424
**RBCs**	4.4 (0.9)	4.2 (0.9)	0.5917
**Platelets**	295.8 (119.0)	355.7 (189.7)	0.3642
**Lymphocytes**	2.37 (1.23)	2.44 (0.92)	0.8760
**Monocytes**	0.58 (0.29)	0.68 (0.24)	0.3674
**Neutrophils**	6.32 (1.38)	6.57 (1.21)	0.6417
**Eosinophils**	0.3 (0.29)	0.25 (0.3)	0.6821
**Basophils**	0.07 (0.08)	0.07 (0.04)	1.0000

**Table 2:** The effect of atorvastatin treatment on the miRNA’s expression array among hematological malignancy patients (n = 12). A negative ΔΔCT (ΔCT_post_—ΔCT_pre_) indicates higher miRNA expression post-treatment. *p*-values were calculated using the Wilcoxon Signed-Rank test for mean difference (ns: not significant, **: *p* < 0.005, and ****: *p* < 0.0001). Abbreviations—ET: Essential Thrombocythemia, CML: Chronic Myeloid Leukemia.

Patient ID	Disease	ΔΔCT	*p*-Value	Significance
**Pt1**	ET	−0.39	0.1605	ns
**Pt2**	ET	−4.22	<0.0001	****
**Pt3**	ET	0.52	0.1179	ns
**Pt4**	ET	−2.02	<0.0001	****
**Pt5**	Lymphoma	1.49	<0.0001	****
**Pt6**	Lymphoma	−2.17	<0.0001	****
**Pt7**	Lymphoma	−4.57	<0.0001	****
**Pt8**	Lymphoma	−2.15	<0.0001	****
**Pt9**	CML	−1.34	0.001	**
**Pt10**	CML	1.79	0.0008	**
**Pt11**	CML	−0.05	0.2062	ns
**Pt12**	CML	−1.29	<0.0001	****

**Table 3:** Pre- and post-atorvastatin treatment expression of statistically significant differentially expressed miRNAs (fold change ≥ 2 or ≤ 0.5 with a two-sided *p*-value ≤ 0.05) in included patients (n = 12). Thirteen miRNAs met this cut-off point, with all being upregulated post-treatment except for one (mir-150), which was downregulated post-treatment. Two-sided *p*-values were calculated using a paired sample *t* test.

	Pre-Treatment ΔCT		Post-Treatment ΔCT				
	Mean	SD	Mean	SD	Fold Change	Trend	*p*-Value
**miR-198**	−0.43	3.66	−4.03	2.70	12.10	Up regulation	0.0062
**let-7-family**	−0.21	2.48	−3.48	5.49	9.64	Up regulation	0.0557
**miR-29a+b+c**	1.71	2.57	−1.42	2.43	8.77	Up regulation	0.0060
**miR-204**	−2.09	3.54	−5.17	3.00	8.44	Up regulation	0.0015
**miR-222**	−0.37	2.66	−2.98	2.48	6.09	Up regulation	0.0194
**miR-224**	4.71	3.45	2.24	2.67	5.53	Up regulation	0.0060
**miR-155**	0.62	2.42	−1.69	3.04	4.97	Up regulation	0.0209
**miR-128b**	3.50	2.85	1.20	2.99	4.96	Up regulation	0.0442
**miR-296**	−1.09	2.87	−2.99	2.38	3.75	Up regulation	0.0459
**miR-199a+b**	0.61	2.82	−1.24	2.48	3.61	Up regulation	0.0493
**miR-194**	0.03	1.91	−1.78	2.67	3.51	Up regulation	0.0439
**miR-125a**	−5.26	1.73	−7.04	2.39	3.44	Up regulation	0.0148
**miR-200a**	1.41	2.30	0.11	2.26	2.46	Up regulation	0.0112
**miR-150**	−6.44	3.03	−4.20	3.35	0.21	Down regulation	0.0500

**Table 4:** Involved genes in enriched KEGG pathways for predicted target gene sets of differentially expressed miRNAs.

miRNA	Pathway	Fold Enrichment	Benjamini *p*-Value	Involved Genes
**mir-198**	hsa05202:Transcriptional misregulation in cancer	27.46	0.0038	CCND2, SIX4, MET, PBX1
**let-7a**	hsa04115:p53 signaling pathway	9.48	0.0256	CDKN1A, RRM2, CCND2, CCND1, MDM4, THBS1
**let-7a**	hsa05219:Bladder cancer	12.91	0.0256	CDKN1A, NRAS, CCND1, E2F2, THBS1
**mir-29a**	hsa04510:Focal adhesion	8.35	0.0001	CDC42, ITGB1, COL3A1, COL1A2, CCND2, LAMA2, COL4A1, AKT3, COL5A2, ITGA6
**mir-29a**	hsa04151:PI3K-Akt signaling pathway	5.98	0.0001	ITGB1, COL3A1, COL1A2, CCND2, LAMA2, COL4A1, AKT3, COL5A2, ITGA6, FOXO3, SGK1, MCL1
**mir-29a**	hsa04512:ECM-receptor interaction	13.84	0.0003	ITGB1, COL3A1, COL1A2, LAMA2, COL4A1, COL5A2, ITGA6
**mir-29a**	hsa05222:Small cell lung cancer	10.12	0.0377	ITGB1, LAMA2, COL4A1, AKT3, ITGA6
**mir-29b**	hsa04151:PI3K-Akt signaling pathway	7.75	0.0000	LAMA2, LAMC1, COL1A1, COL3A1, COL2A1, COL5A1, COL4A1, PDGFC, COL5A2, AKT3, COL4A6, COL4A5, COL6A3, ITGA6
**mir-29b**	hsa05222:Small cell lung cancer	15.74	0.0001	LAMA2, COL4A1, AKT3, COL4A6, COL4A5, ITGA6, LAMC1
**mir-29b**	hsa05200:Pathways in cancer	4.86	0.0015	TGFB2, LAMA2, COL4A1, AKT3, STAT3, COL4A6, COL4A5, ITGA6, VHL, LAMC1
**mir-29c**	hsa04151:PI3K-Akt signaling pathway	4.98	0.0379	COL1A1, COL3A1, COL1A2, AKT3, COL5A2, ITGA6, MCL1
**mir-204**	hsa04022:cGMP-PKG signaling pathway	10.51	0.0017	PPP1CC, PPP3R1, CREB1, ITPR1, CACNA1C, ATP2B1, CREB5
**mir-204**	hsa04922:Glucagon signaling pathway	11.98	0.0220	PPP3R1, CREB1, ITPR1, SIRT1, CREB5
**mir-204**	hsa04024:cAMP signaling pathway	7.19	0.0224	PPP1CC, CREB1, BDNF, CACNA1C, ATP2B1, CREB5
**mir-204**	hsa04550:Signaling pathways regulating pluripotency of stem cells	8.47	0.0242	ZFHX3, DVL3, JAK2, JARID2, BMPR1A
**mir-128b**	hsa04068:FoxO signaling pathway	9.78	0.0016	SMAD2, G6PC3, PDPK1, SETD7, PIK3R1, MAPK14, SOS1, TGFBR1
**mir-128b**	hsa05223:Non-small cell lung cancer	14.62	0.0217	RXRA, PDPK1, E2F3, PIK3R1, SOS1
**mir-128b**	hsa04910:Insulin signaling pathway	7.12	0.0306	G6PC3, PDPK1, PDE3B, MKNK2, PIK3R1, SOS1
**mir-199a**	hsa05200:Pathways in cancer	7.00	0.0025	IKBKB, GSK3B, TGFB2, FZD4, E2F3, WNT2, ETS1, HIF1A
**mir-29b**	hsa04512:ECM-receptor interaction	26.36	0.0000	COL1A1, COL3A1, COL2A1, LAMA2, COL5A1, COL4A1, COL5A2, COL4A6, COL4A5, COL6A3, ITGA6, LAMC1
**mir-29b**	hsa04510:Focal adhesion	12.99	0.0000	LAMA2, LAMC1, COL1A1, COL3A1, COL2A1, COL5A1, COL4A1, PDGFC, COL5A2, AKT3, COL4A6, COL4A5, COL6A3, ITGA6
**mir-155**	hsa04550:Signaling pathways regulating pluripotency of stem cells	8.93	0.0513	SMAD2, SMAD1, MEIS1, ZIC3, KRAS, SMAD5
**mir-128b**	hsa04550:Signaling pathways regulating pluripotency of stem cells	7.02	0.0306	SMAD2, ZFHX3, BMPR2, PIK3R1, MAPK14, ISL1
**let-7a**	hsa05206:MicroRNAs in cancer	5.18	0.0002	TRIM71, CDKN1A, ITGB3, RDX, HMGA2, DICER1, THBS1, NRAS, SOCS1, CCND2, CCND1, IGF2BP1, E2F2, MDM4

## Data Availability

The authors will provide the primary research data included in this manuscript upon request.

## Supplementary Materials

The following supporting information can be downloaded at: https://www.mdpi.com/article/10.3390/biom14121559/s1. Table S1: Oncogenic miRNA array consisting of 95 miRNAs involved in tumorigenesis, apoptosis, and differentiation. Table S2. Pre and post atorvastatin treatment expression of the thirteen statistically significant differentially expressed. Table S3. Fold change for statistically insignificant differentially expressed miRNA pre and post treatment in hematological malignancy patients (n = 12). Table S4: Involved genes in enriched GO biological processes for predicted target gene sets of differentially expressed miRNAs. Table S5. Involved genes in enriched GO cellular components for predicted target gene sets of differentially expressed miRNAs. Table S6. Involved genes in enriched GO molecular functions for predicted target gene sets of differentially expressed miRNA.

## References

[1] Swerdlow S.H., Campo E., Harris N.L., Jaffe E.S., Pileri S.A., Stein H., Thiele J. (2008). WHO Classification of Tumours of the Haematopoietic and Lymphoid Tissues.

[2] Lichtman M.A. (2008). Battling the hematological malignancies: The 200 years’ war. Oncologist.

[3] Siegel R.L., Miller K.D., Jemal A. (2016). Cancer statistics, 2016. CA Cancer J. Clin..

[4] Bachireddy P., Burkhardt U.E., Rajasagi M., Wu C.J. (2015). Haematological malignancies: At the forefront of immunotherapeutic innovation. Nat. Rev. Cancer.

[5] Im A., Pavletic S.Z. (2017). Immunotherapy in hematologic malignancies: Past, present, and future. J. Hematol. Oncol..

[6] Hamilton A., Gallipoli P., Nicholson E., Holyoake T.L. (2010). Targeted therapy in haematological malignancies. J. Pathol..

[7] Ma H., Mallampati S., An G., Wang J. (2015). Targeted Therapy in Hematological Malignancies: From Basic Research to Clinical Practice. BioMed Res. Int..

[8] Patlak M. (2002). Targeting leukemia: From bench to bedside. FASEB J. Off. Publ. Fed. Am. Soc. Exp. Biol..

[9] Terwey T.H., Kim T.D., Arnold R. (2009). Allogeneic hematopoietic stem cell transplantation for adult acute lymphocytic leukemia. Curr. Hematol. Malig. Rep..

[10] Boiron J.M., Cony-Makhoul P., Mahon F.X., Pigneux A., Puntous M., Reiffers J. (1994). Treatment of hematological malignancies relapsing after allogeneic bone marrow transplantation. Blood Rev..

[11] Raguz S., Yagüe E. (2008). Resistance to chemotherapy: New treatments and novel insights into an old problem. Br. J. Cancer.

[12] Bockorny B., Dasanu C.A. (2015). HMG-CoA reductase inhibitors as adjuvant treatment for hematologic malignancies: What is the current evidence?. Ann. Hematol..

[13] Gazzerro P., Proto M.C., Gangemi G., Malfitano A.M., Ciaglia E., Pisanti S., Santoro A., Laezza C., Bifulco M. (2012). Pharmacological actions of statins: A critical appraisal in the management of cancer. Pharmacol. Rev..

[14] Markowska A., Antoszczak M., Markowska J., Huczyński A. (2020). Statins: HMG-CoA Reductase Inhibitors as Potential Anticancer Agents against Malignant Neoplasms in Women. Pharmaceuticals.

[15] Zhou Q., Liao J.K. (2009). Statins and cardiovascular diseases: From cholesterol lowering to pleiotropy. Curr. Pharm. Des..

[16] Beckwitt C.H., Brufsky A., Oltvai Z.N., Wells A. (2018). Statin drugs to reduce breast cancer recurrence and mortality. Breast Cancer Res..

[17] Barbalata C.I., Tefas L.R., Achim M., Tomuta I., Porfire A.S. (2020). Statins in risk-reduction and treatment of cancer. World J. Clin. Oncol..

[18] Yi X., Jia W., Jin Y., Zhen S. (2014). Statin use is associated with reduced risk of haematological malignancies: Evidence from a meta-analysis. PLoS ONE.

[19] Pradelli D., Soranna D., Zambon A., Catapano A., Mancia G., La Vecchia C., Corrao G. (2015). Statins use and the risk of all and subtype hematological malignancies: A meta-analysis of observational studies. Cancer Med..

[20] Bonetti P.O., Lerman L.O., Napoli C., Lerman A. (2003). Statin effects beyond lipid lowering-are they clinically relevant?. Eur. Heart J..

[21] Eulalio A., Huntzinger E., Izaurralde E. (2008). Getting to the root of miRNA-mediated gene silencing. Cell.

[22] Peng Y., Croce C.M. (2016). The role of MicroRNAs in human cancer. Signal Transduct. Target. Ther..

[23] Di Leva G., Garofalo M., Croce C.M. (2014). MicroRNAs in cancer. Annu. Rev. Pathol..

[24] Ventura A., Jacks T. (2009). MicroRNAs and cancer: Short RNAs go a long way. Cell.

[25] Khan I.A., Rashid S., Singh N., Rashid S., Singh V., Gunjan D., Das P., Dash N.R., Pandey R.M., Chauhan S.S. (2021). Panel of serum miRNAs as potential non-invasive biomarkers for pancreatic ductal adenocarcinoma. Sci. Rep..

[26] Zhao A., Zhou H., Yang J., Li M., Niu T. (2023). Epigenetic regulation in hematopoiesis and its implications in the targeted therapy of hematologic malignancies. Signal Transduct. Target. Ther..

[27] Tsai H.P., Huang S.F., Li C.F., Chien H.T., Chen S.C. (2018). Differential microRNA expression in breast cancer with different onset age. PLoS ONE.

[28] Wu Y., Wei J., Zhang W., Xie M., Wang X., Xu J. (2020). Serum Exosomal miR-1290 is a Potential Biomarker for Lung Adenocarcinoma. OncoTargets Ther..

[29] Shi Y., Zhuang Y., Zhang J., Chen M., Wu S. (2020). Identification of Tumorigenic and Prognostic Biomarkers in Colorectal Cancer Based on microRNA Expression Profiles. BioMed Res. Int..

[30] Gablo N., Trachtova K., Prochazka V., Hlavsa J., Grolich T., Kiss I., Srovnal J., Rehulkova A., Lovecek M., Skalicky P. (2020). Identification and Validation of Circulating Micrornas as Prognostic Biomarkers in Pancreatic Ductal Adenocarcinoma Patients Undergoing Surgical Resection. J. Clin. Med..

[31] Xie L., Jing R., Qi J., Lin Z., Ju S. (2015). Drug resistance-related microRNAs in hematological malignancies: Translating basic evidence into therapeutic strategies. Blood Rev..

[32] Abdi J., Jian H., Chang H. (2016). Role of micro-RNAs in drug resistance of multiple myeloma. Oncotarget.

[33] Si W., Shen J., Zheng H., Fan W. (2019). The role and mechanisms of action of microRNAs in cancer drug resistance. Clin. Epigenetics.

[34] Peixoto da Silva S., Caires H.R., Bergantim R., Guimarães J.E., Vasconcelos M.H. (2022). miRNAs mediated drug resistance in hematological malignancies. Semin. Cancer Biol..

[35] Allegra A., Alonci A., Campo S., Penna G., Petrungaro A., Gerace D., Musolino C. (2012). Circulating microRNAs: New biomarkers in diagnosis, prognosis and treatment of cancer (Review). Int. J. Oncol..

[36] Han Z., Rosen S.T., Querfeld C. (2020). Targeting microRNA in hematologic malignancies. Curr. Opin. Oncol..

[37] Chaplin M., Kirkham J.J., Dwan K., Sloan D.J., Davies G., Jorgensen A.L. (2020). STrengthening the Reporting Of Pharmacogenetic Studies: Development of the STROPS guideline. PLoS Med..

[38] Livak K.J., Schmittgen T.D. (2001). Analysis of relative gene expression data using real-time quantitative PCR and the 2(-Delta Delta C(T)) Method. Methods.

[39] Sherman B.T., Hao M., Qiu J., Jiao X., Baseler M.W., Lane H.C., Imamichi T., Chang W. (2022). DAVID: A web server for functional enrichment analysis and functional annotation of gene lists (2021 update). Nucleic Acids Res..

[40] The Cancer Genome Atlas Research Network (2013). Genomic and Epigenomic Landscapes of Adult De Novo Acute Myeloid Leukemia. N. Engl. J. Med..

[41] IBM Corp (2019). IBM SPSS Statistics for Windows.

[42] GraphPad Software (2021). GraphPad Prism.

[43] Boudreau D.M., Yu O., Johnson J. (2010). Statin use and cancer risk: A comprehensive review. Expert Opin. Drug Saf..

[44] Zeichner S., Mihos C.G., Santana O. (2012). The pleiotropic effects and therapeutic potential of the hydroxy-methyl-glutaryl-CoA reductase inhibitors in malignancies: A comprehensive review. J. Cancer Res. Ther..

[45] Jakobisiak M., Golab J. (2003). Potential antitumor effects of statins (Review). Int. J. Oncol..

[46] Mohajeri M., Banach M., Atkin S.L., Butler A.E., Ruscica M., Watts G.F., Sahebkar A. (2018). MicroRNAs: Novel Molecular Targets and Response Modulators of Statin Therapy. Trends Pharmacol. Sci..

[47] Mishra S., Yadav T., Rani V. (2016). Exploring miRNA based approaches in cancer diagnostics and therapeutics. Crit. Rev. Oncol. Hematol..

[48] Stiegelbauer V., Perakis S., Deutsch A., Ling H., Gerger A., Pichler M. (2014). MicroRNAs as novel predictive biomarkers and therapeutic targets in colorectal cancer. World J. Gastroenterol..

[49] Ben-Shushan D., Markovsky E., Gibori H., Tiram G., Scomparin A., Satchi-Fainaro R. (2014). Overcoming obstacles in microRNA delivery towards improved cancer therapy. Drug Deliv. Transl. Res..

[50] Zhang Y., Wang Z., Gemeinhart R.A. (2013). Progress in microRNA delivery. J. Control. Release Off. J. Control. Release Soc..

[51] Witten L., Slack F.J. (2020). miR-155 as a novel clinical target for hematological malignancies. Carcinogenesis.

[52] Russ A.C., Sander S., Lück S.C., Lang K.M., Bauer M., Rücker F.G., Kestler H.A., Schlenk R.F., Döhner H., Holzmann K. (2011). Integrative nucleophosmin mutation-associated microRNA and gene expression pattern analysis identifies novel microRNA—Target gene interactions in acute myeloid leukemia. Haematologica.

[53] Jones S. (2004). An overview of the basic helix-loop-helix proteins. Genome Biol..

[54] Li T., Pan H., Li R. (2016). The dual regulatory role of miR-204 in cancer. Tumor Biol..

[55] Zhao M., Mishra L., Deng C.X. (2018). The role of TGF-β/SMAD4 signaling in cancer. Int. J. Biol. Sci..

[56] Shi Y., Massagué J. (2003). Mechanisms of TGF-beta signaling from cell membrane to the nucleus. Cell.

[57] Connolly E.C., Freimuth J., Akhurst R.J. (2012). Complexities of TGF-β targeted cancer therapy. Int. J. Biol. Sci..

[58] Narayan N., Morenos L., Phipson B., Willis S.N., Brumatti G., Eggers S., Lalaoui N., Brown L.M., Kosasih H.J., Bartolo R.C. (2017). Functionally distinct roles for different miR-155 expression levels through contrasting effects on gene expression, in acute myeloid leukaemia. Leukemia.

[59] Ishihara K., Sasaki D., Tsuruda K., Inokuchi N., Nagai K., Hasegawa H., Yanagihara K., Kamihira S. (2012). Impact of miR-155 and miR-126 as novel biomarkers on the assessment of disease progression and prognosis in adult T-cell leukemia. Cancer Epidemiol..

[60] Due H., Schönherz A.A., Ryø L., Primo M.N., Jespersen D.S., Thomsen E.A., Roug A.S., Xiao M., Tan X., Pang Y. (2019). MicroRNA-155 controls vincristine sensitivity and predicts superior clinical outcome in diffuse large B-cell lymphoma. Blood Adv..

[61] Stamatopoulos B., Van Damme M., Crompot E., Dessars B., El Housni H., Mineur P., Meuleman N., Bron D., Lagneaux L. (2015). Opposite Prognostic Significance of Cellular and Serum Circulating MicroRNA-150 in Patients with Chronic Lymphocytic Leukemia. Mol. Med..

[62] Zhou H., Li Y., Liu B., Shan Y., Li Y., Zhao L., Su Z., Jia L. (2017). Downregulation of miR-224 and let-7i contribute to cell survival and chemoresistance in chronic myeloid leukemia cells by regulating ST3GAL IV expression. Gene.

[63] Seca H., Lima R.T., Almeida G.M., Sobrinho-Simoes M., Bergantim R., Guimaraes J.E., Vasconcelos M.H. (2014). Effect of miR-128 in DNA damage of HL-60 acute myeloid leukemia cells. Curr. Pharm. Biotechnol..

