# Supplementary material for: The Effect of Atorvastatin on Oncogenic miRNAs in Hematological Malignancies: A Central Study
Source: Biomolecules. 2024 Dec 7;14(12):1559. doi: 10.3390/biom14121559 (PMC11673652; doi:10.3390/biom14121559)
Supplement: Supplementary file 1 [file biomolecules-14-01559-s001.zip › biomolecules-3291432-supplementary.pdf]

biomolecules

MDPI

Supplementary Materials

The Effect of Atorvastatin on Oncogenic miRNAs in Hematological Malignancies: A Central Study

Jood Hashem [1,*,†], Farah Alsukhni [1,†], Hassan Abushukair [2] and Mahmoud Ayesh [2]

[1] Department of Medical Laboratory Sciences, Jordan University of Science and Technology, Irbid, Jordan
[2] Faculty of Medicine, Jordan University of Science and Technology, Irbid, Jordan
* Correspondence: jmhashem@just.edu.jo; Tel.: +962-79-9932316
† These authors contributed equally to this work.

1. Molecular Study Details

1.1. RNA Extraction from Plasma Sample

The miRNeasy Serum/Plasma Kit (Qiagen, Germany) was used for miRNA purification. The procedure was performed using a standard phenol/guanidine-based technology combined with silica-membrane purification using miRNeasy mini spin columns. A 200 μL plasma sample was first lysed using Trizol, followed by chloroform-three phase separation with buffering conditions (salts and pH) that allow the binding of only RNA to the miRNeasy spin column membrane. This was followed by washing steps using 2 different washing buffers; RWT buffer and RPE buffer, to completely remove residual contaminants and improve the purity of the eluted miRNAs. Finally, purified miRNAs were eluted with RNase-free water. The concentration of the eluted RNA and its purity were spectrophotometrically measured using an ND-2000 Nanodrop (Thermo Scientific, MA, USA). Purified miRNAs samples were stored at − 80 °C for long term-storage.

1.2. cDNA Synthesis

For all samples, we performed miRNAs normalization to 30 ng/mL, then miRNAs were reverse transcribed to cDNA using the QuantiMi Kit (System Biosciences, CA,USA). This included three steps: tagging all small RNAs with a poly-A tail, annealing an oligo-dT adaptor, and reverse transcription to create first-strand cDNA, using a thermocycler (Applied Biosystems). The result was a cDNA pool from anchor-tailed miRNAs that are ready for real-time PCR. The concentration and the purity of the synthesized cDNA were measured by ND-2000 Nanodrop (Thermo Scientific, MA, USA), and stored at –20 °C for further analysis.

1.3. miRNAs Quantification by Real-Time qPCR

Circulating miRNAs were quantified by real-time quantitative PCR using an Applied Biosystems 7500 Fast-well system (Applied Biosystems, Warrington, United Kingdom). The quantitative assay was performed by using OncoMir qPCR Array (System Biosciences, CA,USA) based on amplification of 95 miRNAs involved in tumorigenesis, apoptosis, and differentiation, carefully selected from published literature (Table S1). Specific miRNAs primer sequences and the universal miRNAs sequences were previously designed by the manufacture.

1.4. Real-time qPCR Reaction Setup:

For qPCR, the reaction mixture for an entire 96-well plate included 1.750 μL 2 × SYBR Green (qPCR mastermix buffer), 60 μL Universal Reverse Primer (10 μM), 20 μL sample; synthesized cDNA, and 1,670 μL RNase-free water. From the reaction mixture, 29 μL were put per well in the qPCR plate. The plate was then covered with an optical adhesive cover and spun briefly (800 × g /for 1 min at cold temperature). The final step was to load 1 μL

per well of each of the primers, from the primer plate into the qPCR plate (well A1 into qPCR plate A1, etc.) The guidelines as detailed for specific Real-time instrumentation were followed, including a melt analysis after the qPCR run to assess the Tm of the PCR amplicon to verify the specificity of the amplification reaction. The Cycle thresholds CT were set within the exponential phase of the amplification plots with software automatic baseline settings. qPCR cycling and data accumulation conditions were as follows: pre-holding stage at 50 °C for 2 min, holding stage at 95 °C for 10 min, cycling stage; denaturation step at 95 °C for 15 sec, and finally cycling stage; annealing step at 60 °C for 1 min. The number of cycles was 40 cycles for steps 3 and 4, and data read was at 60 °C for 15 sec as mentioned in the array user manual. The relative expression level difference between the four groups in our study (pre and post Atorvastatin administration for both HM patient and control subjects was done by comparing cycle threshold CT values (2-ddCT)

1.5. Exosomes Precipitation

For exosome isolation a specific precipitation technique was used based on using a solution containing a polymer that can bind to the CD63; an exosome specific marker, resulting in gently precipitating exosomes between 30 and 200 nm in size from serum. 24 First, Serum samples were centrifuged at low speed for 15 min to remove cells and cellular debris, then an appropriate amount of ExoQuick precipitation solution was added to the cleared serum, and then the serum was refrigerated for 30 min at 4 °C. After incubation, ExoQuick/serum mixture was centrifuged at 1500 × g for 30 min at 4 °C. then, the supernatant was aspirated, and the residual solution was re-spun down by light centrifugation at 1500 × g for 5 min for ensure complete removal of all traces of fluid by aspiration. Finally, exosome pellet was suspended in 150 µL using sterile 1 × DPBS, a recommended buffer for the next ELISA quantification assay.

1.6. Exosome Quantification by ELISA

Circulating plasma exosomes were quantified by ELISA using ExoELISA-ULTRA Complete Kit (CD63 detection). Exosome concentrations were measured using a sandwich ELISA kit purchased from System Biosciences, USA. According to manufacturer instruction, isolated exosome samples were diluted in 150 µL of 1 × DPBS. Absorbance was measured spectrophotometrically at 450 nm using an 800™ TS Microplate reader

Table S1. Oncogenic miRNA array consisting of 95 miRNAs involved in tumorigenesis, apoptosis, and differentiation.

1	2	3	4	5	6	7	8	9	10	11	12
et-7 family	miR-7	miR-92	miR-93	miR-9-1	miR-101	miR-103	miR-106a	miR-106b	miR-107	miR-10b	miR-1
miR-122a	miR-125a	miR-125b	miR-126	miR-128b	miR-132	miR-133a	miR-134	miR-135b	miR-136	miR-137	miR-140
miR-141	miR-142-3p	miR-143	miR-145	miR-146a	miR-149	miR-150	miR-151	miR-153	miR-154	miR-155	miR-15a
miR-15b	miR-16	miR-17-3p	miR-17-5p	miR-181a	miR-181b	miR-181c	miR-181d	miR-183	miR-185	miR-186	miR-188
miR-190	miR-191	miR-192	miR-194	miR-195	miR-196a	miR-197	miR-198	miR-199a+b	miR-30b	miR-19a+b	miR-18a
miR-95	miR-20a	miR-200a	miR-200b	miR-200c	miR-202	miR-203	miR-204	miR-205	miR-206	miR-21	miR-210
miR-214	miR-215	miR-372	miR-373	miR-218	miR-219	miR-22	miR-488	miR-221	miR-222	miR-223	miR-224
miR-23a	miR-24	miR-25	miR-26a	miR-26b	miR-27a+b	miR-30c	miR29a+b+c	miR-30a-3p	miR-30a-5p	miR-296	snRNA U6

Table S2. Pre and post atorvastatin treatment expression of the thirteen statistically significant differentially expressed.

	Pretreatment ΔCT		Posttreatment ΔCT			
	**Mean**	**SD**	**Mean**	**SD**	**Fold Change**	**P value**
**miR-198**	-1.03	5.70	-0.92	3.46	0.93	0.9732
**let-7-family**	2.08	2.56	0.84	2.89	2.36	0.3929
**miR-29a+b+c**	0.39	3.17	0.72	1.80	0.79	0.8328
**miR-204**	-3.02	4.56	-2.88	3.23	0.91	0.9622
**miR-222**	-0.39	3.66	0.29	1.44	0.62	0.7625
**miR-224**	3.41	3.04	2.55	3.11	1.81	0.6638
**miR-155**	-0.21	2.65	-0.46	3.35	1.19	0.9350
**miR-128b**	1.08	3.07	2.71	2.31	0.32	0.5414
**miR-296**	-1.56	5.62	-3.01	5.60	2.74	0.4888
**miR-199a+b**	1.78	2.44	0.80	2.74	1.97	0.6782
**miR-194**	-0.57	3.34	-0.19	2.11	0.77	0.8909
**miR-125a**	-7.23	4.59	-6.49	1.66	0.59	0.7863
**miR-200a**	-0.05	1.88	3.73	2.97	0.07	0.1239
**miR-150**	-5.82	3.35	-3.91	2.06	0.27	0.3260

Table S3. Fold change for statistically insignificant differentially expressed miRNA pre and post treatment in hematological malignancy patients (n = 12).

	Fold Change	*P* value
**miR-136**	7.67	0.0794
**miR-206**	5.89	0.1218
**miR-185**	5.46	0.0686
**miR-145**	5.09	0.1688
**miR-16**	4.48	0.1357
**miR-30a-5p**	4.23	0.0732
**miR-488**	4.08	0.0622
**miR-221**	3.98	0.1511
**miR-210**	3.86	0.1067
**miR-373**	3.79	0.0573
**miR-19a+b**	3.72	0.1592
**miR-214**	3.48	0.0721
**miR-195**	3.46	0.0847
**miR-215**	3.27	0.5047
**miR-7**	3.16	0.1859
**miR-134**	3.10	0.2074
**miR-137**	3.06	0.2200
**miR-186**	3.01	0.1400
**miR-22**	2.94	0.2840
**miR-21**	2.90	0.1364
**miR-200b**	2.78	0.2863
**miR-197**	2.64	0.2252
**miR-9-1**	2.60	0.1344
**miR-141**	2.58	0.1982
**miR-205**	2.48	0.3106
**miR-30a-3p**	2.47	0.2160
**miR-188**	2.40	0.2528
**miR-203**	2.36	0.2098
**miR-218**	2.34	0.1737
**miR-202**	2.30	0.3453
**miR-143**	2.28	0.3617
**miR-27a+b**	2.18	0.2131
**miR-10b**	2.16	0.2731
**miR-17-5p**	2.09	0.1905
**miR-142-3p**	2.05	0.3226
**miR-106a**	1.98	0.2577
**miR-126**	1.96	0.1670
**miR-93**	1.96	0.0546
**miR-200c**	1.91	0.4303

**miR-223**	1.87	0.4682
**miR-191**	1.83	0.4742
**miR-106b**	1.83	0.4913
**miR-24**	1.82	0.4720
**miR-92**	1.82	0.0525
**miR-25**	1.82	0.5093
**miR-30b**	1.79	0.4415
**miR-196a**	1.78	0.4213
**miR-1-1**	1.73	0.4442
**miR-101-1**	1.73	0.2540
**miR-149**	1.54	0.6580
**miR-125b**	1.54	0.3658
**miR-103**	1.53	0.5671
**miR-192**	1.51	0.4844
**miR-219**	1.47	0.6804
**miR-181d**	1.41	0.4645
**miR-23a**	1.41	0.6421
**miR-190**	1.41	0.5797
**miR-95**	1.36	0.6764
**miR-372**	1.36	0.6111
**miR-151**	1.33	0.7833
**miR-15b**	1.30	0.7147
**miR-122a**	1.30	0.6098
**miR-181a**	1.29	0.7567
**miR-181c**	1.25	0.7810
**miR-132**	1.24	0.5222
**miR-133a**	1.22	0.8119
**miR-26b**	1.21	0.8294
**miR-135b**	1.16	0.8316
**miR-20a**	1.08	0.9138
**miR-15a**	0.98	0.9833
**miR-30c**	0.96	0.9617
**miR-140**	0.93	0.9190
**miR-17-3p**	0.88	0.8643
**miR-107**	0.82	0.8005
**miR-183**	0.80	0.8025
**miR-146a**	0.50	0.5725
**miR-26a**	0.43	0.4560
**miR-181b**	0.40	0.4177
**miR-154**	0.39	0.4282
**miR-18a**	0.35	0.2419

Table S4: Involved genes in enriched GO biological processes for predicted target gene sets of differentially expressed miRNAs.

miRNA	Pathway	Fold Enrichment	Benjamini *P* value	Involved Genes
**mir-125a**	GO:0097192~extrinsic apoptotic signaling pathway in absence of ligand	52.92	0.0000	ERBB3, MKNK2, BCL2, CASP2, BAK1, MCL1
**mir-128b**	GO:0032869~cellular response to insulin stimulus	15.39	0.0170	PDPK1, SP1, PDE3B, PIK3R1, ZFP36L1, WDTC1
**mir-155**	GO:0006366~transcription from RNA polymerase II promoter	5.54	0.0071	SMAD2, SMAD1, MEIS1, ZIC3, TRPS1, FOS, HIVEP2, HIF1A, BACH1, MYBL1, RREB1
**mir-155**	GO:0007179~transforming growth factor beta receptor signaling pathway	14.04	0.0378	SMAD2, SMAD1, FOS, CBL, SMAD5
**mir-199a**	GO:0001837~epithelial to mesenchymal transition	68.12	0.0074	GSK3B, TGFB2, SNAI1, HIF1A
**mir-199a**	GO:0045944~positive regulation of transcription from RNA polymerase II promoter	5.90	0.0074	IKBKB, GSK3B, DDX3X, CCNL1, ACVR1B, WNT2, ETS1, HIF1A, SIRT1, JUNB
**mir-200a**	GO:0045944~positive regulation of transcription from RNA polymerase II promoter	6.52	0.0301	YAP1, KLF12, ZEB2, ZEB1, GATA6, OGT, FOXA2, IRF2BPL
**mir-204**	GO:0045944~positive regulation of transcription from RNA polymerase II promoter	3.84	0.0324	CCNT2, TCF12, HMGA2, NR3C1, SIRT1, MEIS2, ARNTL2, PPP3R1, CREB1, TCF4, SOX4, BCL9L, BMPR1A
**mir-204**	GO:0000122~negative regulation of transcription from RNA polymerase II promoter	4.42	0.0326	ZFHX3, HNRNPA2B1, WWC3, ZBTB20, HMGA2, TCF4, EZR, JARID2, SIRT1, MEIS2, HMX1
**mir-222**	GO:1900740~positive regulation of protein insertion into mitochondrial membrane involved in apoptotic signaling pathway	53.31	0.0245	BCL2L11, TP53BP2, BMF, BBC3
**mir-29a**	GO:0030198~extracellular matrix organization	10.86	0.0010	ITGB1, COL3A1, COL1A2, LAMA2, COL4A1, ELN, COL5A2, ITGA6, FBN1
**mir-29b**	GO:0030198~extracellular matrix organization	19.35	0.0000	LAMA2, LAMC1, COL1A1, COL3A1, COL2A1, COL5A1, COL4A1, COL7A1, COL5A2, COL4A6, COL4A5, COL6A3, ITGA6, FBN1

**mir-29c**	GO:0080111~DNA demethylation	57.25	0.0986	TDG, TET3, TET2

Table S5. Involved genes in enriched GO cellular components for predicted target gene sets of differentially expressed miRNAs.

miRNA	Pathway	Fold Enrichment	Benjamini *P* value	Involved Genes
**mir-29a**	GO:0005654~nucleoplasm	2.40	0.0033	CCNT2, DOT1L, FOXO3, CAND1, CCND2, TDG, AKT3, VHL, MCL1, KDM6B, CBX6, SETDB1, FEM1B, KMT5C, DNMT3A, ZBTB34, KLF4, MORF4L1, REST, NASP, NFIA, IMPDH1, AGO1, REL, HBP1, SGK1
**mir-29a**	GO:0031012~extracellular matrix	6.94	0.0111	COL3A1, COL1A2, LAMA2, COL4A1, COL5A2, LPL, ADAMTS9, FBN1
**mir-29b**	GO:0005604~basement membrane	29.29	0.0000	COL2A1, LAMA2, COL5A1, COL4A1, COL7A1, ITGA6, LAMC1, FBN1
**mir-29b**	GO:0005578~proteinaceous extracellular matrix	6.48	0.0393	COL5A1, COL5A2, COL4A6, COL4A5, COL6A3, FBN1
**mir-155**	GO:0005667~transcription factor complex	12.68	0.0000	SMAD2, SMAD1, MEIS1, TRPS1, TCF4, FOS, HIF1A, SMAD5, RCOR1
**mir-155**	GO:0071141~SMAD protein complex	102.00	0.0091	SMAD2, SMAD1, SMAD5
**mir-125a**	GO:0005730~nucleolus	4.33	0.0155	ESRRA, LIN28B, NIN, LIN28A, DHX33, TRAF6, MKNK2, ARID3A, TACC2, SIRT7, ZNF385A

Table S6. Involved genes in enriched GO molecular functions for predicted target gene sets of differentially expressed miRNA.

miRNA	Pathway	Fold Enrichment	Benjamini *P* value	Involved Genes
**let-7c**	GO:0001205~transcriptional activator activity, RNA polymerase II distal enhancer sequence-specific binding	28.37	0.0039	MEF2C, PBX3, HMGA1, ARID3B, BACH1
**let-7c**	GO:0000980~RNA polymerase II distal enhancer sequence-specific DNA binding	13.09	0.0093	MEF2C, SMARCC1, MBD2, PBX3, ARID3B, BACH1
**mir-29a**	GO:0005201~extracellular matrix structural constituent	21.00	0.0009	COL3A1, COL1A2, COL4A1, ELN, COL5A2, FBN1

**mir-29b**	GO:0048407~platelet-derived growth factor binding	118.05	0.0000	COL1A1, COL3A1, COL2A1, COL5A1, COL4A1
**mir-29c**	GO:0048407~platelet-derived growth factor binding	85.26	0.0279	COL1A1, COL3A1, COL1A2
**mir-204**	GO:0001077~transcriptional activator activity, RNA polymerase II core promoter proximal region sequence-specific binding	9.70	0.0032	CREB1, TCF12, HMGA2, TCF4, NR3C1, MEIS2, SOX4, RUNX2
**mir-204**	GO:0043425~bHLH transcription factor binding	49.76	0.0067	TCF12, TCF4, SIRT1, RUNX2
**mir-155**	GO:0003700~transcription factor activity, sequence-specific DNA binding	3.73	0.0041	SMAD2, SMAD1, RORA, FOS, CBL, HIF1A, SMAD5, BACH1, ZIC3, TRPS1, CREBRF, TCF4, HIVEP2, RREB1
**mir-155**	GO:0030618~transforming growth factor beta receptor, pathway-specific cytoplasmic mediator activity	153.46	0.0071	SMAD2, SMAD1, SMAD5
**mir-155**	GO:0019901~protein kinase binding	5.44	0.0189	SMAD1, SOCS1, ADAM10, PTPRJ, RICTOR, RAB11FIP2, HIF1A, YWHAZ
**mir-155**	GO:0008134~transcription factor binding	6.30	0.0189	SMAD2, RORA, FOS, HIF1A, YWHAZ, RCOR1, SMARCA4
**mir-155**	GO:0000979~RNA polymerase II core promoter sequence-specific DNA binding	17.95	0.0299	SMAD1, FOS, SMAD5, RREB1
